# Systematic comparison of HIV-1 Envelope-specific IgG responses induced by different vaccination regimens: Can we steer IgG recognition towards regions of viral vulnerability?

**DOI:** 10.3389/fimmu.2022.1075606

**Published:** 2023-01-09

**Authors:** Augusta Horvath, Lisa Rogers, Georgios Pollakis, Olga Baranov, Nora Pieroth, Sarah Joseph, Mkunde Chachage, Asli Heitzer, Lucas Maganga, Frank Msafiri, Agricola Joachim, Edna Viegas, Leigh-Anne Eller, Hannah Kibuuka, Supachai Rerks-Ngarm, Punnee Pitisuttithum, Sorachai Nitayapan, Jittima Dhitavat, Nakorn Premsri, Sarah Fidler, Robin J. Shattock, Merlin Lee Robb, Jonathan Weber, Sheena McCormack, Patricia Jane Munseri, Eligius Lyamuya, Charlotta Nilsson, Arne Kroidl, Michael Hoelscher, Ralf Wagner, Christof Geldmacher, Kathrin Held

**Affiliations:** ^1^ Division of Infectious Diseases and Tropical Medicine, University Hospital, LMU Munich, Munich, Germany; ^2^ German Center for Infection Research (DZIF), Partner Site Munich, Munich, Germany; ^3^ Institute of Infection Veterinary and Ecological Sciences (IVES/CIMI), University of Liverpool, Liverpool, United Kingdom; ^4^ MRC Clinical Trials Unit at UCL, Institute of Clinical Trials and Methodology, University College London, London, United Kingdom; ^5^ National Institute for Medical Research–Mbeya Medical Research Centre (NIMR-MMRC), Mbeya, Tanzania; ^6^ Muhimbili University of Health and Allied Sciences, Dar es Salaam, Tanzania; ^7^ Instituto Nacional de Saúde, Maputo, Mozambique; ^8^ United States Military HIV Research Program, Silver Spring, MD, United States; ^9^ Makerere University Walter Reed Project, Kampala, Uganda; ^10^ Henry M. Jackson Foundation for the Advancement of Military Medicine, Bethesda, MD, United States; ^11^ Department of Disease Control, Ministry of Public Health, Mueang Nonthaburi, Thailand; ^12^ Faculty of Tropical Medicine, Mahidol University, Bangkok, Thailand; ^13^ Armed Forces Research Institute of Medical Science, Bangkok, Thailand; ^14^ Department of Medicine, Imperial College London, London, United Kingdom; ^15^ Department of Laboratory Medicine, Karolinska Institute, Huddinge, Sweden; ^16^ The Public Health Agency of Sweden, Solna, Sweden; ^17^ Institute of Medical Microbiology and Hygiene, University Regensburg, Regensburg, Germany; ^18^ Institute of Clinical Microbiology and Hygiene; University Hospital Regensburg, Regensburg, Germany

**Keywords:** HIV, CN54rgp140 vaccine, envelope-specific antibodies, immunogen sequence, V3-antibodies, V2-antibodies, linear peptide array, vaccine

## Abstract

Immunogens and vaccination regimens can influence patterns of immune-epitope recognition, steering them towards or away from epitopes of potential viral vulnerability. HIV-1 envelope (Env)-specific antibodies targeting variable region 2 (V2) or 3 (V3) correlated with protection during the RV144 trial, however, it was suggested that the immunodominant V3 region might divert antibody responses away from other relevant sites. We mapped IgG responses against linear Env epitopes in five clinical HIV vaccine trials, revealing a specific pattern of Env targeting for each regimen. Notable V2 responses were only induced in trials administering CRF01_AE based immunogens, but targeting of V3 was seen in all trials, with the soluble, trimeric CN54gp140 protein eliciting robust V3 recognition. Strong V3 targeting was linked to greater overall response, increased number of total recognised antigenic regions, and where present, stronger V2 recognition. Hence, strong induction of V3-specific antibodies did not negatively impact the targeting of other linear epitopes in this study, suggesting that the induction of antibodies against V3 and other regions of potential viral vulnerability need not be necessarily mutually exclusive.

## Introduction

There is still an urgent need for a sufficiently protective HIV vaccine. Enormous genetic variability, high viral mutation rates leading to escape variants, and poor surface accessibility of the HIV-1 Env protein are major hurdles to the elicitation of a protective immune response (1–3). The HIV-1 virus is always “one step ahead of its host”, and viral variants that successfully evade the human immune response typically prevail. An HIV vaccine might be able to break this vicious cycle (4).

The holy grail of HIV vaccine research remains the induction of broadly neutralizing antibodies (bNAb) with the ability to neutralise a wide range of viral variants. Despite substantial progress in this field, induction of broad cross-neutralizing antibodies by vaccination still remains challenging (1, 5). In addition, the very few people living with HIV who produce highly potent bNAb do so only after several years of exposure to HIV (6, 7). On the other hand, vaccine-induced non-neutralizing antibodies (nNAb) to the HIV-1 Env, the immune correlate of reduced risk of HIV infection in the RV144 trial, appear to be a more achievable goal.

RV144, the only efficacy trial to date in which at least moderate protection against HIV acquisition was achieved (8, 9), was conducted between 2003 and 2009 in 16,402 volunteers in Thailand in the context of a HIV-1 subtype CRF01_AE dominated epidemic. A modified intention to treat analysis showed an estimated overall efficacy of 60.5% at 12 months after the first vaccination (10), which waned to 31.2% 3.5 years later (8). Binding IgG antibodies to specific linear epitopes of the HIV-1 Env variable regions 2 (V2) and 3 (V3) correlated inversely with HIV-1 infection in RV144, whereas neutralizing antibodies were not associated with a reduction in infection risk (9, 11, 12). Envelope sequence analyses of breakthrough infections confirmed the selective pressure of V2-specific antibody responses in the RV144 trial (13–15) and further studies showed a parallel decline of vaccine efficacy and the level of anti-V2 IgG responses over time (12, 16–18). During natural infection, antibodies against the highly variable V2 region are found in less than 50% of infected individuals (19, 20) while anti-V3 antibodies can be found in almost all naturally HIV-1 infected individuals and are elicited by most vaccination regimen tested so far (18, 21–27). The V3 sequence is the most conserved of all the variable Env regions (28) and important for the pathogenicity of the virus (29). The protective potential of V3-specific antibodies is further supported by the association of maternal anti-V3 nNAb with a reduced mother-to-child transmission (30, 31).

Contrary to these promising findings on the role of nNAb, it has been hypothesized that their immunodominance, especially of V3-directed nNAb, is to blame for the great difficulty in inducing bNAb (32).The rationale behind this is that in the germinal centre B cells with high affinity to such immunodominant epitopes as V3, strongly activate and recruit T follicular helper cells (Tfh) and might have a selection advantage over bNAb B cell precursors with lower affinity (33). Particularly, if Tfh help is limited and nNAb and bNAb epitopes are in competition, V3-responses may outcompete the maturation of weaker-affinity binding antibodies, necessary for bNAb formation (32). Accordingly, there is an effort in vaccine development to remove or repress the highly immunogenic V3 epitope to eliminate such potential decoy effects, in hopes of inducing bNAb against the HIV-1 Env (34, 35). There are currently many ingenious vaccination strategies under investigation to guide antibody affinity maturation towards the development of bNAbs (1), a formidable challenge that will, despite initial success (36), not be achieved in the foreseeable future. Regions of putative viral vulnerability, including V2 and V3, should therefore continue to be regarded as key target regions for a protective HIV vaccine. The overall objective of this study therefore was i) to investigate how different prime boost vaccination regimen in multiple clinical vaccine trials influence the pattern of IgG Env recognition; ii) to investigate vaccine parameters influencing IgG targeting of HIV-1 Env V2 and V3 epitopes and their sequence variants; and iii) to understand whether strong induction of V3-specific IgG responses compromises the recognition of other antigenic regions. To this end, we systematically mapped HIV-1 Env IgG epitopes from multiple HIV vaccine studies (RV144, TaMoVac02, UKHVC Spoke003, X001, and RV172) to identify immunogens and their combinations for optimal induction of responses to (36)wards regions of putative viral vulnerability.

## Results

### Different prime boost vaccination regimens induce distinct patterns of IgG Env recognition

**Figure 1:**
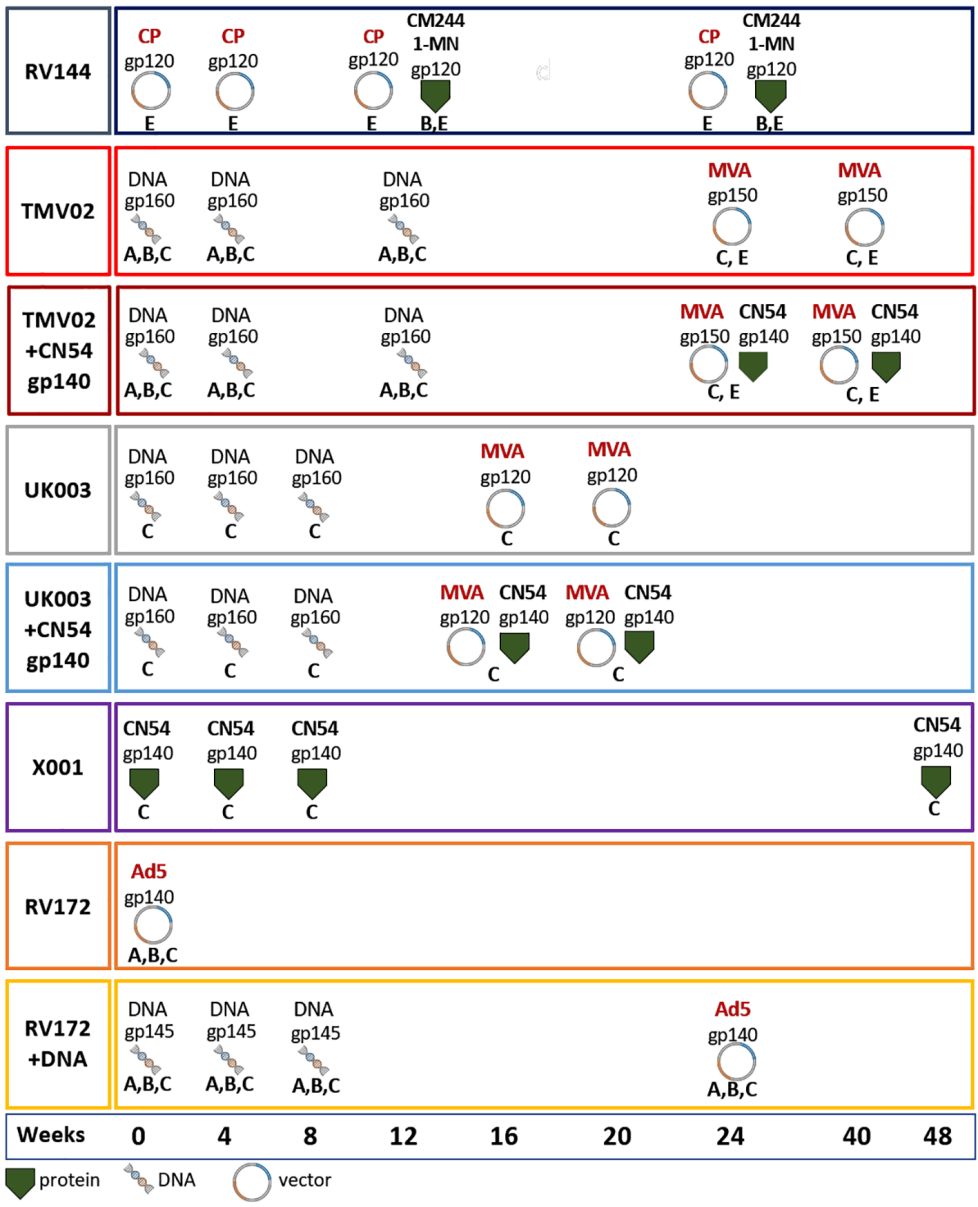
Vaccination schedules. Vaccination schedules and immunogens of the eight vaccination groups from five different HIV vaccine trials analysed herein. For each vaccination group the molecular forms as well as the HIV-1 clade of the Env immunogens, their delivery form, and time point of administration are stated. The immunogens included: gp120 monomers, gp140 soluble trimers, open trimeric structures, membrane anchored gp145, ‘native-like’ gp150 including the transmembrane region and native trimeric gp160. Immunogens were either administered as adjuvanted proteins or expressed *in vivo* using DNA, MVA, CP and/or Ad5. Most groups received prime-boost vaccine regimens including multiple sequence variants of the Env (RV144, TMV02, RV172), while some included only CN54 derived immunogens (UK003, X001). The colour coding of the surrounding frame for each vaccination group is kept consistently in this article. Viral Vectors: MVA, Modified Vaccinia Ankara; CP, Canary Pox; Ad5, Adenovirus 5; Proteins: CM244, CRF01_AE strain; 1-MN = B-strain, CN54 = C-strain.

**Figure 2:**
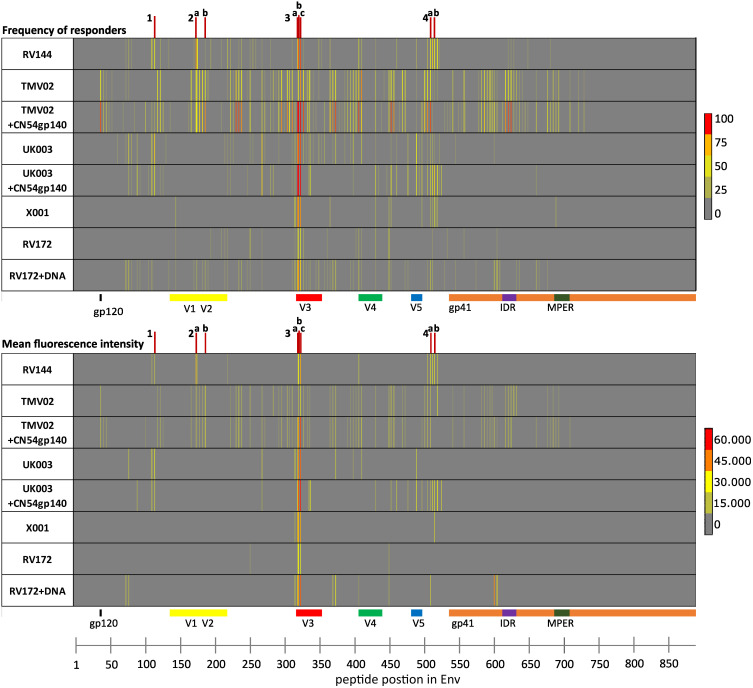
Analysis of IgG epitope recognition along the HIV-1 Env protein in the 8 different vaccine groups. The frequency of responders (FOR; upper panel) and the mean fluorescence intensities (mean FI; lower panel) plotted against individual antigenic regions along the entire HIV-1 Env as included in the 10 full-length Env immunogen sequences comprising the array backbone. Each row of the respective heat maps displays the Env-specific IgG responses of one of 8 vaccination groups, tested four weeks after the last vaccination. IgG responses against individual antigenic regions were considered positive if the corresponding FI was above 3,500 after subtraction of the pre-vaccination value. The mean FI was calculated using the maximum FI values per position for each participant, only if peptide-specific IgG responses occurred in at least 25% of the vaccinees. IDRs 1–4 are indicated by red lines and are listed in **Table 1**.

Using a linear peptide microarray approach, we systematically mapped the specificity of Env-specific serum IgG responses in individuals from eight distinct vaccination groups (named herein: RV144, n=10; TMV02, n=10; TMV02+CN54gp140, n=10; UK003 n=10; UK003+CN54gp140, n=10; X001, n=5; RV172, n=10; RV172+DNA, n=10; **Figure 1**). The peptide array consisted of 1034 HIV Envelope 15-mer peptides, overlapping by 11 amino acids to cover the whole gp160 extracellular domain, including 10 full-length Env immunogen sequences, previously identified highly immunogenic regions and frequently in the HIV sequence database occurring antigenic variants, covering all HIV-1 subtypes. A more detailed description of the array is provided in **Supplementary Figure 1** and the methods section. A detailed description of vaccination schedules and study groups is given in **Figure 1** and the Methods section. Each of these HIV-1 vaccine trials induced a unique pattern of IgG Env recognition. **Figure 2** gives an overview of these different linear B-cell epitopes along the HIV-1 Env for the respective groups.

Several frequently and strongly targeted Env epitopes were identified. Of these, four immunodominant regions (IDRs) were detected (lines in **Figure 2**). IDRs were defined by a frequency of responders (FOR) of at least 60% and with a mean fluorescence intensity (FI) value within the top 15% of all FI values in at least one vaccination group. These IDRs were located within gp120 and are listed in **Table 1**. FOR and mean FI values for each IDR and vaccination group can be found in Supplementary Table 2.

**Table 1:** Summary of immunodominant antigenic regions (IDR’s).

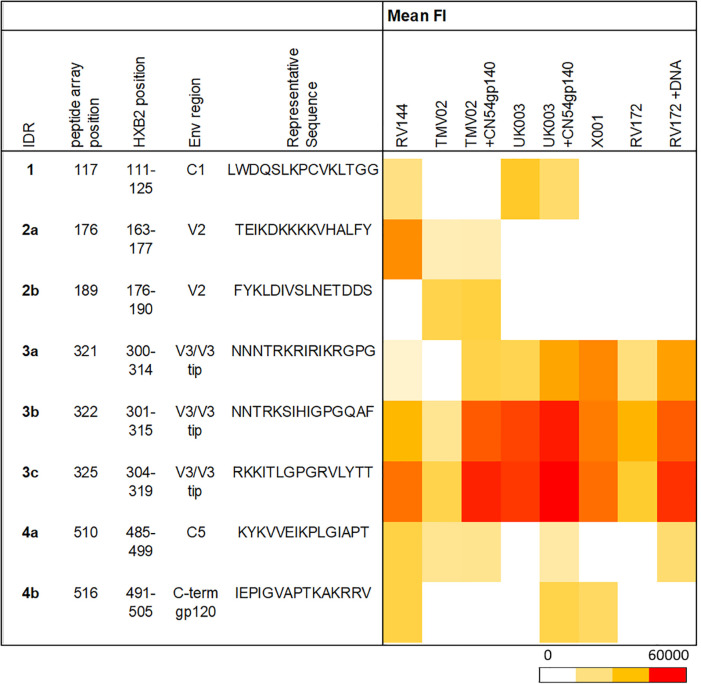	

IDRs were defined by a detection frequency of at least 60% responders and with mean FI values in the top 15% of all peptides in all groups. One region corresponds to one peptide of 15 consecutive amino acids. For each IDR, the position on the array, the corresponding HXB2 position, and a representative sequence are listed. IDRs with overlapping peptides were named with the same number and distinguished from each other by different letters. Further, the mean FI for each IDR is depicted as a heat map per vaccination group. Mean FI values were only calculated if the FOR was ≥25%. IDRs 1-4 are indicated by red lines in **Figure 2**. FOR and mean FI values for each trial can be found in Supplementary Table 2. IDR, immunodominant region; FOR, frequency of Responders; mean FI, mean fluorescence intensity.

The first IDR is located within the constant region one of gp120 (C1), designated IDR1_C1 (HXB2_111-125). C1 specific recognition was mainly induced in the RV144 trial and both subgroups of the UK003 trial (**Figure 2**, **Table 1**). The next IDRs were found within the V2 region: IDR2a_V2 (HXB2_163-177) and IDR2b_V2 (HXB2_176-190). Recognition of the IDR2a_V2 was only detected in RV144 and TMV02 participants and IDR2b_V2 was primarily recognised by participants in the TMV02 trial (**Figure 2**, **Table 1**). Within the V3 region IDR3a_V3 (HXB2_300-314), IDR3b_V3 (HXB2_301-315) and IDR3c_V3 (HXB2_304-319), three largely overlapping IDRs, were induced in all HIV-1 vaccine trials. Of these, the response to IDR3c_V3 was strongest (**Figure 2**, **Table 1**). Constant region five (C5) contained two overlapping IDRs, designated as IDR4a_C5 (HXB2_485-499) and IDR4b_C5 (HXB2_491-505). IDR_C5a was recognised in all groups, except RV172. IDR4b_C5 was induced only by the vaccination regimens of the RV144, UK003+CN54gp140 and the X001 trials (**Figure 2**, **Table 1**).

In summary, different vaccination regimens led to the induction of antibodies targeting various HIV-1 Env IgG epitopes, with four prominent IDRs. These distinct patterns now provide us with a unique opportunity to compare the 8 different selected vaccine groups in a side-by-side analysis and allow us to study the impact of certain immunogen sequences or HIV-1 Env molecular forms on the Env specific IgG recognition pattern.

### Gp120 immunogens in UK003 and RV144 induced frequent recognition of conserved regions

**Figure 3:**
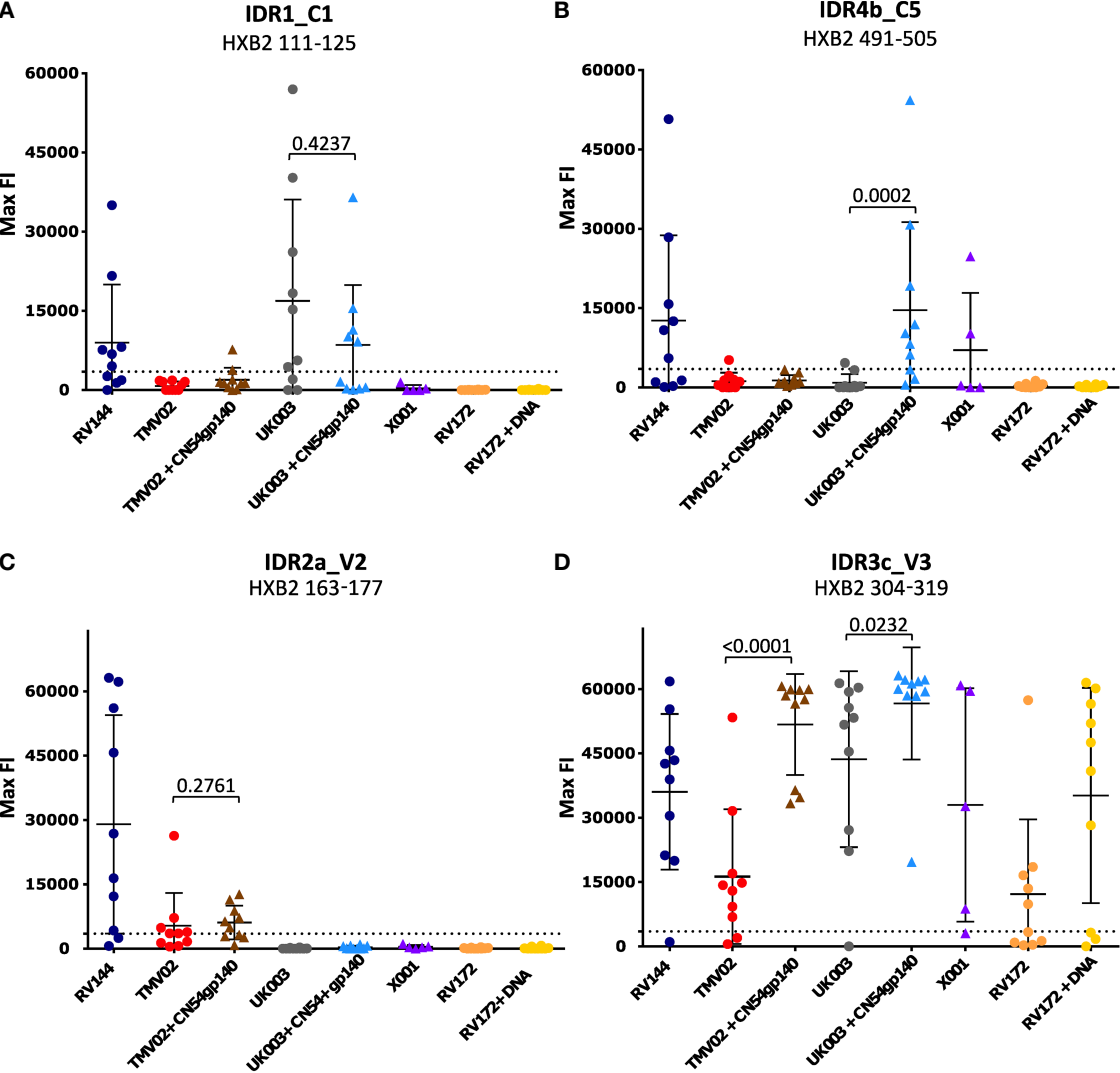
Statistical comparison of IgG responses targeting single peptide IDRs. Graphs **(A-D)** each depict the statistical comparison of the FI values of one representative peptide of the 4 IDRs. Each symbol indicates the maximum FI value of one single study participant. Values after subtraction of the baseline are shown. The cut-off for positive signals is indicated by a dotted line. P-values were calculated using a Mann-Whitey-U test. P-values of comparisons between groups with or without CN54gp140 protein boosts are shown. Triangular symbols indicate that the CN54gp140 protein was part of the study’s vaccination schedule, round symbols represent vaccinees that did not receive the CN54gp140 protein.

Comparison of the patterns of Env recognition across the study groups revealed that IDR1_C1 recognition was mainly induced by the RV144 trial and both UK003 vaccination regimens; the only trials using gp120 monomeric immunogens. To control for the impact of CN54gp140 we formally compared the IDR1_C1 response in the two subgroups of the UK003 trial, which differed solely by the administration of this protein (**Figure 3A**). There was no significant difference in the level or frequency of detection of the C1 region between the UK003 vaccination groups (p=0.4237). There was also no significant difference in the detection of these C1 epitopes between the UK003 and RV144 participants (RV144 vs. UK003+CN54gp140: p=0.8928; RV144 vs. UK003: p=0.5779) despite the different vaccination components (**Figure 3A**). To a minor extent, this can also be observed for IDR4a_C5 and b_C5, as only vaccination regimen including the gp120 immunogens (RV144 and UK003+CN54gp140) induced strong and frequent responses against both of these epitopes (**Table 1**), without significant difference between IDR4b_C5 responses in RV144 vs. UK003+CN54gp140 vaccinees (p=0.5678) (**Figure 3B**). Yet for IDR4_C5 there seems to be an impact of CN54gp140, as for the UK003 trial C5 directed recognition was only elicited in the CN54gp140 boosted group compared to the standard UK003 group. Hence, strong and frequent recognition of conserved regions C1 and C5 were mainly induced by the two vaccine trials that included gp120 immunogens (UK003 and RV144), confirming previous results (12, 25).

### V2 targeting in the RV144 and the TMV02 trial is linked to the administration of a particular CRF01_AE V2 sequence

A closer look at the V2 Env epitope, previously associated with protection in the RV144 trial (9, 12), revealed that this epitope, here deemed IDR2a_V2 (HXB2_163-177) was targeted exclusively and with high frequency by RV144 and TMV02 vaccinees (**Figure 2**, **Table 1**).

IDR2a_V2 directed IgG responses were stronger in RV144 compared to TMV02 (RV144 vs. TMV02: p=0.0232; RV144 vs. TMV02+CN54gp140: p=0.0749) (**Figure 3C**). Maximum FI (max FI) values of the individual participants in RV144 showed a considerable spread (range: 619-63121; mean = 28989), but were overall at a high level and in some participants even reached the upper detection limit. The max FI values of IDR2a_V2 in both TMV02 groups were generally lower (for TMV02+CN54gp140, range: 4914-12646; mean: 6088 and for TMV02, range: 3562-26312; mean = 5343). No significant difference of max FI values could be detected between the two TMV02 subgroups (p=0.2761, **Figure 3C**). This argues against a negative influence of CN54gp140 on the elicitation of V2 IgG responses.

In our study RV144 and TMV02 are the only trials that use immunogens based on clade CRF01_AE. In RV144 the clade E sequence was presented *via* gp120 encoded in a Canary Pox (CP) vector as well as part of the bivalent gp120 CM244 protein. For both TMV02 groups this sequence was present in the Modified Vaccinia Ancara (MVA) encoded gp150. For all these immunogens the underlying amino acid sequence representing the V2 region was identical (Supplementary Table 3). Other vaccination parameters, including dosage form, molecular Env structure (gp120 vs gp150), and the remaining immunogens differed.

**Figure 4:**
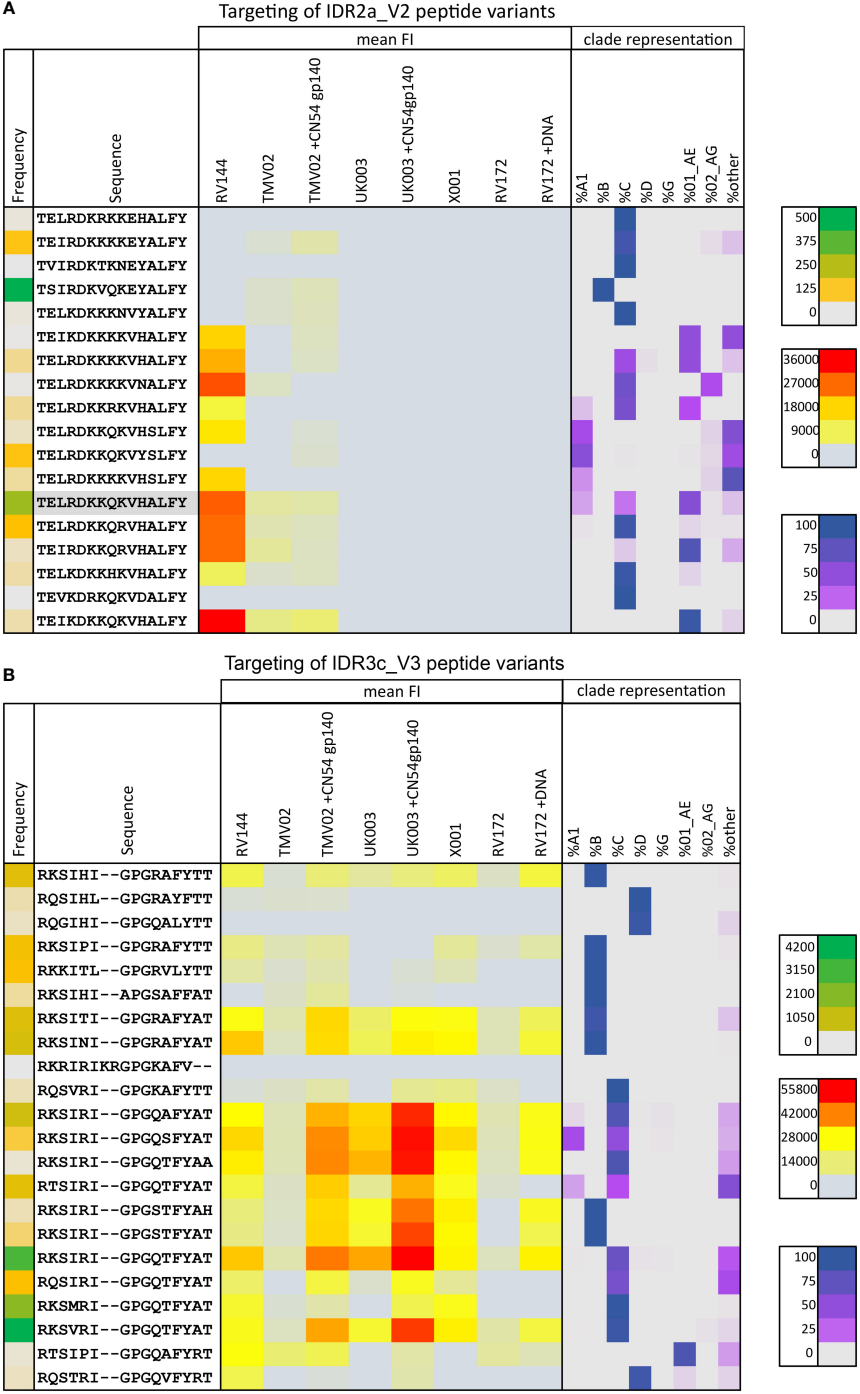
Targeting of IDR2a_V2 and IDR3c_V3 peptide variants by vaccine-induced antibodies. The mean magnitude of antibody responses against the respective peptide variant was calculated per group if positive responses occurred in >25% of vaccinees above background (3500 FI) after baseline subtraction. Mean FI values for each vaccination group (yellow to red) are illustrated as a heat map in the context of their frequency of occurrence in the HIV database (green coding on the left), and their clade representation (purple coding on the right). Green colour-coding symbolises the frequency of the respective peptide in the Los Alamos (www.hiv.lanl.gov) database representing the current global HIV epidemic, varying from grey (low) to green (high) according to the prevalence of the peptide. Red colour coding represents the magnitude of the IgG response towards each given peptide. The distribution of occurrences of a peptide variant within HIV-1 clades as a rounded fraction is depicted in purple. **(A)** Heat map of 18 peptide variants corresponding to the HXB163_TGMIDKMKEEYALFY V2 position. The CRF01_AE immunogen sequence is highlighted in grey. **(B)** 22 peptide variants were included for the V3 tip region (HXB304_RKSIRIGPGSTFYAT). Additional peptide variants were included in the peptide microarray to cover a broad range of variants and responses in these specific Env areas of interest.

Focusing on the different peptide variants in the peptide microarray representing this region, the highest recognised variants were common to both studies and closely related to the CRF01_AE immunogen sequence (**Figure 4A**). All variants detected in RV144 deviated from this sequence by a maximum of 2 amino acids and contained the amino acids K169 and V172. The variants most strongly detected in TMV02, also contained 169K, whereas those deviating more from the vaccine sequence, showed only FI levels close to background.

These data suggest that this CRF01_AE immunogen sequence has the potential to generate the IDR2a_V2 specific V2 (HXB2_163-177) response.

### The CN54gp140 protein induces strong V3-specific IgG responses

Across all the studies we included, the strongest and most frequent IgG response was directed against the V3 region. A closer look at this highly immunogenic region and the vaccination regimen, revealed that the strongest response to V3 seen in participants from the UK003+CN54gp140 group (**Figure 3D**, IDR3c_V3: mean FI=56637), followed by the TMV02+CN54gp140 group (mean FI=51734). Participants of both trials received the same CN54gp140 protein based on the sequence described before (37, 38). This protein was co-administered with either MVA-C-gp120 (UK003) or a MVA-E-gp150 after priming with DNA-C-gp160 (UK003) or DNA-A_B_C-gp160 (TMV02) (**Figure 1**). For both trials we observed that the subgroups without the CN54gp140 protein boost had significantly weaker V3 responses (UK003+CN54gp140 vs. UK003, p=0.0232; TMV02+CN54gp140 vs. TMV02, p<0.0001; **Figure 3D**). The homologous DNA-C-gp120 prime and MVA-C-gp120 boost in the UK003 group resulted in very strong V3 recognition (**Figure 3D**: mean FI=43640). Of note, all immunogens in the UK003 study were based on the same clade C CN54 sequence. In contrast, V3 recognition in the TMV02 group was among the weakest of our compared groups (**Figure 3C**, mean FI = 16257).

Administration of the CN54gp140 protein alone in the X001 trial, led to a strong mean response, but substantial interindividual variability was observed (**Figure 3D**, mean FI=32971). Generally, the anti-Env response in X001, induced by 4 vaccination with CN54gp140, had a strong focus on V3, with few other epitopes being recognized (**Figure 2**), confirming previous results (39). RV144 and RV172+DNA vaccinees also showed distinct V3 responses with similar magnitudes (**Figure 3D**, mean FI=36034 and mean FI=35174, respectively). The weakest V3 recognition was found in the RV172 group, lacking the DNA priming (**Figure 3C**, mean FI=12204).

Moreover, the V3-directed IgG response showed strong cross-variant and cross-clade reactivity (**Figure 4B**). For all vaccination studies analysed here, we see a similar pattern of variant recognition, albeit at varying intensities, with very high recognition magnitudes in the TMV02+CN54gp140 group and the UK003+CN54gp140 group. Overall, the degree of cross-recognition of V3 was higher compared to V2 and highest in those who received a V3 sequence homologous DNA prime and CN45gp140 boost (**Figures 4A, B**), confirming previous results (25, 26).

In summary, the V3 region was a main target across all trial groups, and the CN54gp140 protein proved to induce or boost highly robust and cross-reactive IgG recognition of this region.

### Strong induction of V3-specific IgG responses did not negatively affect the targeting of other antigenic regions

**Figure 5:**
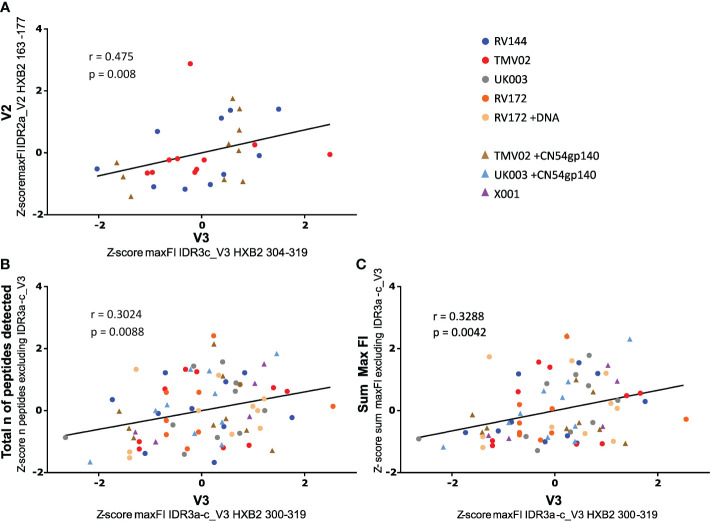
Impact of V3 detection on V2 tip and total Env reactivity. Scatter plots depict the relationship between the strength of V3 IgG recognition and **(A)** V2 detection, **(B)** the total number of peptides detected, and **(C)** overall Env detection. Data were Z normalised (mean of 0, standard deviation of 1) to allow comparison of all trials. A Spearman correlation analysis was used to calculate the statistical relationship. **(A)** Correlation of the intensity of IDR3c_V3 IgG targeting with IDR2a_V2 recognition. For the scaled data, the r-value was: 0.475, the p-value was: 0.008 and the 95% CI calculated by bootstrapping was 0.1273 to 0.7187. V2_IDR2a and V3_IDR3c are represented by one single peptide each. Only data of study participants from studies with both responses (RV144 and TMV02) are plotted. **(B)** Correlation of the FI values of the whole IDR3_V3 with the total number of Env peptides detected excluding the IDR3_V3. The r-value was: 0.3024, the p-value was: 0.0088 and the 95% CI calculated by bootstrapping was 0.07250 to 0.5018. **(C)** Correlation of the FI values of the whole IDR3_V3 with the total Env detection strength bar the IDR3_V3. The observed r-value was: 0.3288, the p-value was: 0.0042 and the 95% CI calculated by bootstrapping was 0.1016 to 0.5234. In **(B)** and **(C)** the max FI values for the three IDRs belonging to V3 (IDR3a-c) were summed up per patient. The individual studies are differentiated by colour, with each symbol representing one participant. The line indicates the linear fit of the data. Participants whose regimen included CN54gp140 are symbolised by triangles, all others by dots.

It has been hypothesised that the strong induction of V3-specific antibody recognition may compromise the vaccine induced recognition of other antigenic regions (32, 34, 35). To address this, we asked the question of whether strong V3 responses correlated negatively with IgG recognition of the linear V2 epitope associated with protection in RV144 (IDR2a_V2 HXB2 163-177) or overall Env recognition. A Spearman rank correlation after Z-normalization of all max FI values (**Figure 5**), demonstrated a positive, linear correlation between the recognition strength of V3 (HXB2_304) and V2 (HXB2_163) in RV144 and TMV02 study participants (r=0.475, p-value=0.008, **Figure 5A**). Importantly, there was also no significant difference in the detection of IDR2a_V2 (HXB2_163-177) between the two TMV02 subgroups, regardless of the CN54gp140 protein enhanced V3 response (p=0.2761, **Figure 3B**). Testing the relationship between V3 detection (IDR3a-c) and the total number of detected Env epitopes excluding V3 (IDR3a-c) in all trials (**Figure 5B**), showed a positive linear association with a weak Spearman correlation (r=0.3024, p-value=0.0088). Accordingly, a weak positive association with a significant positive Spearman rank correlation (r=0.3288, p-value=0.0042, **Figure 5C**) is observed between V3 (IDR3a-c) detection and overall detection intensity in all groups. Further, also in individual vaccinees there was no link between above-average V3 recognition and below-average V2 recognition or below-average overall Env reactivity, or vice versa (**Supplementary Figure 2**).

In summary, our data demonstrates that strong induction of V3-specific IgG recognition did not attenuate antibody responses targeting other linear Env epitopes, rather it weakly correlated with stronger linear V2 detection, increased number of total detected regions and overall biding intensity of linear non-glycosylated epitopes.

### Priming with DNA-gp145 in RV172 caused an increase in overall Env-specific IgG recognition magnitude and epitope breadth

Dissecting the effect of three DNA-gp145 priming injections before a single immunization with a soluble Ad5-A_B_C-gp140 in the RV172 study, revealed that DNA priming was associated with a significant increase in the overall magnitude (p = 0.0070) and epitope breadth (p = 0.039) of the Env-specific IgG response (**Supplementary Figure 3**). Further, a positive effect of DNA priming on V3 detection, although not significant, could be seen (**Figure 3D**, p = 0.0887). In addition, for the DNA primed RV172+DNA group, regions within gp41 were another main target (gp41_HXB2 576-594). Recognition of gp41 generally was only observed in the RV172+DNA and TMV02 vaccine groups (**Figure 2**), the only groups receiving either gp145 or gp150. In contrast, the single administration of Ad5 encoded gp140 proved to be poorly immunogenic, both in terms of the breadth and strength of env specific IgG recognition (**Figure 2**). Overall, a positive effect of DNA priming on the immunogenicity of the RV172 vaccine regimen could be observed.

## Discussion

In this study, we have examined how different prime boost vaccination regimens influenced the pattern of IgG HIV-1 Env epitope recognition in order to identify immunogens and immunogen combinations that induce optimal responses towards regions of potential viral vulnerability. Each vaccination regimen elicited a distinct pattern of Env-specific epitope recognition. Whilst targeting of the V2 region was found only in vaccine groups including the CRF01_AE V2 immunogen sequence in their vaccination regimen, the V3 tip region was strongly recognised region in all groups. Here, the CN54gp140 protein proved to be a strong immunogen to evoke or enhance robust and cross-reactive IgG recognition of the V3 tip. Further, we found that strong V3-specific IgG recognition was not associated with weaker overall immunogenicity to other Env-epitopes, but rather correlated with an increased number of total detected regions and stronger linear V2 recognition.

The 4 main IDRs identified herein, located in the C1, V2, V3, and C5 Env regions, have all already been described in previous studies using peptide microarray approaches (12, 25, 26). The conserved regions C1 and C5 are putatively located at the inter-gp120 and the gp41-Gp120 interface, respectively, and therefore might be structurally more accessible to B cell receptor recognition on monomeric compared to more closed trimeric Env immunogens (25). In line with this, C1 (IDR1_C1) recognition was only observed in groups including gp120 immunogens (UK003 and RV144). Here this effect was, however, less pronounced for the detection of C5 (IDR4b_C5).

Anti-V2 IgG responses, targeting the linear V2 epitope (HXB2 163-177), inversely correlated with HIV infection risk in the RV144 trial (9, 12). Our study confirms previous research (12, 25, 26, 40) that IgG recognition of this linear V2 epitope is induced by CRF01_AE based immunisation regardless of their molecular structure. Our side-by-side analysis shows, that the RV144 regimen induced stronger V2 recognition compared to TMV02, whereas both TMV02 groups generated comparable V2 responses. The differences in V2 recognition between RV144 and TMV02 could be multifold, yet we suggest that they are either related to the boosting effect of the recombinant AIDSVAX protein, or to the different presentation forms of the CRF01_AE sequence. For RV144 both the CP vector encoded gp120 Env and the Env gp120 protein, are based on the CRF01_AE sequence, whereas for TMV02 the CRF01_AE sequence was only present in the MVA encoded gp150 Env protein. Presenting the CRF01_AE sequence in two different delivery forms in RV144 in a total of 4 immunizations, was superior to the two MVA vector-based immunisations in TMV02. Moreover, including only 2 different V2 immunogen sequences in RV144 compared to 4-5 different V2 sequences in TMV02 might favour stronger V2 directed IgG responses. Detailed dissection of these V2 responses regarding cross-clade reactivity presented here, showed, that the strongest recognised V2 variants were common to both studies and closely related to the CRF01_AE immunogen sequence. Interestingly, these recognised V2 variants occur in clade AE sequences and also clade C sequences, thus confirming the results of previous *post hoc* RV144 and TMV02 (and predecessor) analyses, where clade AE, followed by clade C sequence variants were detected best (12, 25, 26, 41). Moreover, we observed that all detected variants in RV144 and also the most strongly detected variants in TMV02, contained the amino acid K at HXB2 position 169, as reported before (12, 26). This is of particular interest, as a sieve analyses conducted in RV144 participants showed that the lack of K at this position was critical to breakthrough infection, and therefore a match between exposed HIV-1 variant and vaccine sequence was associated with protection (15). The identification of vaccine parameters driving IgG recognition towards V2, a putative key region for protection from infection, remains of great value and is being raised again in the wake of the HVTN702 phase 2b/3 trial in South Africa, halted due to a lack of efficacy (42). In line with our findings, the clade C based HVTN100/702 regimen was found to elicit weaker and less cross-reactive IgG responses against the linear V2 epitope than the clade AE/B based RV144/HVTN097 regimen (40, 43). These findings indicate that the CRF01_AE sequence used in RV144 and TMV02 may possess structural properties resulting in superior induction of a V2-directed IgG response, which should be considered for the design of future vaccine studies.

Recognition of linear V3 Env epitopes inversely correlated with infection risk in RV144 in the absence of Env-specific plasma IgA (12). In agreement with former analyses, linear V3 Env epitopes were highly immunogenic in all the vaccine trials analysed here and the CN54gp140 protein immunisation was a particularly good in inducing or boosting highly robust and cross-reactive anti-V3-specific IgG responses (12, 21, 23, 25, 26). Whether such responses might contribute to protection is currently being explored in the phase IIb PrEPVacc trial (RIA2016V-1644, https://www.prepvacc.org/).

Whether strong induction of V3 directed responses is desirable is as of yet unclear, as highly immunogenic epitopes such as V3 have been hypothesised to divert the immune response at the expense of other more desirable antigenic regions such as targets for bnAb precursors (32, 34, 44). However, our data demonstrates that strong induction or boosting of V3-specific IgG responses does not attenuate the antibody responses to other linear Env regions and rather correlates with stronger V2 detection and an increased number of total detected regions. Interestingly, shifting of Env-specific antibody responses away from V3 through concealment or elimination has been achieved, but was not accompanied by an increased production of Tier 2 neutralizing Abs (35, 45). In line, studies into stepwise conformational stabilization of Env trimer immunogens demonstrated a decline of V3 reactivity with increasing trimer stabilization, yet also with declining overall Env immunogenicity and V2 responses^27^. These findings, in combination with our results, suggest that a strong V3 response does not negatively interfere with antibody responses to other Env regions and therefore the pursuit of inducing of bNAbs and binding antibodies against regions of putative viral vulnerability does not have to be mutually exclusive.

For the RV172 trial, using an Ad5-based regimen with or without DNA priming, we observed that Env-specific responses of the Ad5 Env gp140 vector regimen were augmented by priming with multiclade DNA encoded Env gp160, with broader antibody responses against the HIV-1 Env, in line with previous findings (46, 47). Of note, Env sequences encoded in Ad5 and DNA of the RV172 trial largely matched, which likely contributed to the significant priming effect mediated by DNA vaccination observed for recognition of multiple antigenic regions throughout gp120 and gp41. It further has to be mentioned, that testing of the RV172 regimen in the HVTN505 phase IIb efficacy trial in the USA failed to show protection (48).

We report the following limitations of our study: With the peptide micro-array employed only linear non-glycosylated HIV-1 Env epitopes will be detected. Yet, these might still be part of continuous or even discontinuous conformational epitopes. Further, only limited numbers for each vaccination group have been tested, however, the pattern of Env targeting is in-line with earlier publications (23–26) and recognition of the IDRs was consistent between vaccinees.

In summary our comparative side-by-side analysis of selected HIV-1 vaccine trials using a HIV-1 Env peptide microarray showed that responses against the V2 region were mainly induced by V2 AE immunogen sequences, regardless of the molecular form, and that strong recognition of linear V3 epitopes was not associated with a weakening of antibody responses against other linear epitopes. These findings contribute to a better understanding of the influence of different vaccine parameters on the IgG recognition of individual linear Env regions and thus inform future vaccination strategies to steer antibody responses towards regions of potential viral susceptibility.

## Materials and methods

For this study, pre-existing anonymised samples were used. All clinical trials were reviewed and approved by the relevant ethical review boards and all trial participants provided written informed consents before any study procedures were performed. Trials have been registered at the US National Institute of Health under registration numbers RV144: NCT00223080, UKHVC Spoke03: NCT01922284, TaMoVac02: NCT01697007, RV172 trial: NCT00123968 and X001: NCT01966900. The systematic comparison of HIV Envelope antigenic regions targeted by IgG responses induced by different HIV vaccination strategies was further approved by the Ethics Committee of the Ludwig Maximilian University in Munich, Germany.

### HIV vaccine trials and specimen

Plasma or sera samples of baseline and 4 weeks after final vaccination were analysed from the following clinical trials; RV144 (8), UKHVC Spoke003 (24), TaMoVac02 (49), RV172 (50), and X001 (39). Participants of each vaccination group were selected randomly amongst those not HIV infected in the course of the trial. **Figure 1** shows the immunization regimens of the 5 trials and their subgroups. More detailed information on the Env vaccine immunogens, immunogens other than Env (Gag, Pol, Nef), vaccine dosage, and delivery forms are provided in Supplementary Table1. RV144 was included as a positive benchmark. To be able to identify immunogens and immunogen combinations for optimal induction of responses towards regions of putative viral vulnerability, analysed time points and vaccine groups were chosen so that individual subgroups of a study (UK003, TMV02, and RV172) differed only by the administration of a single component.

10 HIV negative participants of the RV144 efficacy trial receiving ALVAC at weeks 0 and 4 followed by two boosts at weeks 12 and 24 with ALVAC in combination with AIDSVAX were selected. ALVAC is a recombinant canarypox vector (vCP1521) expressing a membrane-bound gp120 from strain 92TH023 (CRF01_AE), linked to the transmembrane portion of gp41. ALVAC further encodes for Gag and Pol of HIV-1 MN, subtype B. AIDSVAX is a bivalent gp120 protein immunogen based on subtypes B/E and isolates of strain MN and A244 (8).

The TaMoVac02 trial, a phase 2a clinical trial recruited healthy volunteers in Tanzania and Mozambique (49). We analysed 10 plasma samples from each of the two vaccine arms of TMV02 Group1 (49). Both received three DNA vaccinations at weeks 0, 4 and 12, followed by two boosts with a recombinant modified vaccinia Ankara (MVA) with (TMV02+CN45) or without (TMV02) the recombinant subtype C envelope protein CN54rgp140 at weeks 24 and 40. The DNA-based vaccination consisted of 7 DNA plasmids; 3 encoded for the trimeric envelope gp160 of HIV-1 subtypes A, B and C; the remaining 4 plasmids encoded for Gag A/B, HIV-1 Rev B and a mutated form of reverse transcriptase B (51). The MVA vector encoded for a membrane-anchored trimeric gp150, clade E (CRF01_AE).

We examined 10 samples from each of the two subgroups of the UKHVC003 study (24), a clinical randomised phase 1 vaccine trial, conducted on healthy volunteers in the UK. Participants of both groups initially received DNA-based inoculations at weeks 0, 4 and 8, consisting of a plasmid encoding for a trimeric form of the gp160 envelope protein, as well as a ZM96 plasmid, encoding for a gag-pol-nef fusion protein (24). This was followed by two boosts either with an MVA-C only (UK003) or with MVA-C in combination with a CN54gp140 protein (UK003+CN54gp140) at weeks 16 and 20. MVA-C expresses a secreted form of the CN54gp120 Env and Gag-Pol-Nef polyprotein, clade C (52, 53). The complete vaccination schedule of the UK003 group consisted of two additional boosts at weeks 24 and 40 with CN54gp140, however, here we selected plasma samples taken after the second MVA-C vaccination to determine the effect of the additional administration of the CN54gp140 protein.

The X001 phase 1 trial (39) was conducted on a small group of healthy volunteers in the UK. It tested 4 intramuscular inoculations with a recombinant uncleaved clade C HIV-1 envelope gp140 protein (CN54gp140) (54, 55). In our study, we included samples from 5 participants of group B, receiving injections at weeks 0, 4, 8 and 48. Of note we included 5 samples here because the total group size was <10. The trimeric soluble gp140 CN54 protein is based on the same formulation as the CN54 protein boosts of the respective subgroups of TMV02 and the UK003 trials. Of note, CN54gp140 is an uncleaved, not stabilised, soluble trimer (55) that was found to partly deviate in negative-strain electron microscopy scans and to be susceptible to decay into gp120 and gp41 (56).

The RV172 Phase 1/2 Study tested a recombinant Adenovirus serotype 5 (Ad5) vaccine with or without a prior multiclade HIV-1 DNA plasmid inoculation in HIV-uninfected volunteers in East Africa (50). We examined plasma of 10 participants of RV172 group 2 and RV172 group 5 each. Group 2 (RV172) received a single dose of Ad5 (10^11^ PU/ml). Group 5 participants were primed with DNA at weeks 0, 4 and 8 and boosted with the Ad5 vaccine at week 24 (10^10^PU/ml) (RV172+DNA). The DNA-based vaccine (VRC-HIV-DNA016-00-VP) consisted of 6 closed circular DNA plasmids, 3 of which encoded for trimeric HIV-1 envelope gp145 of clade A, B and C, including the transmembrane domain (57, 58). The other 3 plasmids expressed for HIV-1 Gag, Pol and Nef proteins, clade B. The recombinant adenovirus vector-based (VRC-HIVADV014-00-VP) vaccine encoded synthetic, soluble gp140 versions of clades A, B, and C, as well as the Gag-Pol fusion protein of HIV-1 subtype B (59).

### Peptide microarray design

The peptide microarray used in our study, manufactured by JPT (Berlin, Germany), was designed to map IgG recognition of linear HIV-1 Env regions in preclinical and clinical vaccine studies and has been described previously (27). The array consists of 1034 15mer peptides with an 11 amino acid overlap to cover the whole gp160 extracellular domain of the HIV-1 Env. These peptides covered 10 full-length Env immunogen sequences, the so-called backbone, including CN54gp140_AF286226 (C) and MVA-CMDR_AFJ93253 (CRF01_AE) included in the HIV-1 vaccine trials herein, as well as eight additional sequences of preclinical vaccine candidates of the EHVA consortium (https://ehv-a.eu/), namely 96ZM651_AF286224, BG505_DQ208458, ConC, HKM3, ngp41CM, and unpublished. In addition to the backbone, 15 previously identified immunodominant regions were covered by additional peptide variants (**Supplementary Figure 1A**). Selection for these regions of particular interest was based on mapping data from previous studies of RV144, VAX003 and VAX004 (12) and HIVIS/TaMoVac01/02, X001 and UKHVC (12, 23–26, 39). Within these 15 immunodominant regions, the array also covered the most abundant molecular forms from circulating sequence variants from pre-seroconversion (n=913) and recent (n=723) HIV-infection of 192 subjects, obtained from the HIV database (www.hiv.lanl.gov) (**Supplementary Figure 1B**). Immunogen sequences of the analysed vaccine trials were largely covered by the array, with the more divergent RV172 sequences to a slightly lesser extent. The array covered all HIV-1 clades, though clade C is overrepresented due to the focus on immunogen sequences of the EHVA consortium (**Supplementary Figure 1C, D**).

### Linear Peptide Microarray Mapping of HIV-1 Env-specific IgG responses in vaccinees

Peptide microarrays were used according to the manufacturer’s instructions (www.jpt.com) with minor modifications, as described before (25, 27). The arrays were printed in a 4-well-system and each peptide was printed on the array in triplicates. Slides were blocked (Superblock T20, Thermo Fisher Scientific, Waltham, MA, USA) and then incubated for 2 hours with human plasma or sera at a dilution of 1:100 in blocking buffer. After washing, an anti-human IgG DyLight649(Cy5) secondary antibody (Thermo Fisher Scientific, Waltham, MA, USA) was added to detect IgG antibodies bound to the peptides on the array. Assays for individual participants were conducted for baseline and examination time point at the same test run. Slides were scanned on a GenePix 4000A scanner at 650nm to generate a tiff image file and analysed using GenepixPro 6.0 software (Molecular Devices, San José, CA, USA). After adding the array layout (gal file), encoding the location of each peptide, artefacts were excluded in a manual control step. Results were saved as GenePix Results File, which match each peptide position with the corresponding fluorescence intensity (FI) value. For further analysis of these raw data R studio (version 1.4.1106) and Microsoft Excel were used. First, the mean value of the FI of each triplicate was calculated, excluding outliers. Mean FI values, each corresponding to a 15mer peptide, could then be assigned to an alignment, using a fasta file containing both the sequences of the array and the immunogen sequences of the studied trials. Baseline values were subtracted from post-vaccination time points for the calculation of the frequency of responders and mean FI per group. Mean FI values per study group were calculated for each peptide position, using the strongest recognised variant for each position (maximum FI) and a cut-off of 3500 FI after subtraction of the pre-vaccination value to exclude background. Mean FI values for the whole group were calculated, if more than 25% of the vaccinees showed a response directed against the respective peptide. Immunodominant regions (IDRs) were defined as array positions recognised in at least one vaccination group by 60% or more participants with a mean FI value in the top 15% of all peptides in all groups. The mean magnitude of antibody responses against individual peptide variants was calculated per group if positive responses occurred in >25% of vaccinees above background (3500 FI) after baseline subtraction.

### Statistical analysis

In order to statistically compare the impact of the CN54gp140 protein boost and differences between selected vaccine trials, a Mann-Whitney nonparametric u-test was used. Individual maximum FI values after subtraction of the baseline were used without applying the cut-off of 3500 FI to reflect individual values (**Figure 3** and **Supplementary Figure 3**). P-values < 0.05 were considered significant. A Spearman rank correlation was used to calculate the statistical relationship between responses against V3 and other epitopes. To compare vaccination groups of different orders of magnitude and variances Z normalized values were calculated (mean of 0, standard deviation of 1). R-values between +0.3 and +0.5 were considered as a weak positive correlation. For the relationship of two individual peptides (IDR2a_V2 and IDR3c_V3), maximum FI values after baseline subtraction without cut-off, were used. Considering regions of multiple peptides or overall immunogenicity, maximum FI values after baseline subtraction >3500 (Cut-off) were included (**Figure 5**). Possible associations of above average V3 responses with below average V2 responses were explored by calculating the deviation from the median response per Env epitope for each vaccine (**Supplementary Figure 2**). Graph Pad Prism V6.01 and Python 3.8 were used for statistical analyses.

## Data availability statement

The raw data supporting the conclusions of this article will be made available by the authors, without undue reservation. All relevant data are available in the main text and supplementary information.

## Ethics statement

The studies involving human participants were reviewed and approved by Ethics Committee of the Ludwig Maximilian University in Munich, Germany. The patients/participants provided their written informed consent to participate in this study. For this study, pre-existing anonymised samples were used. All clinical trials were reviewed and approved by the relevant ethical review boards and all trial participants provided written informed consents before any study procedures were performed. The systematic comparison of HIV Envelope antigenic regions targeted by IgG responses induced by different HIV vaccination strategies was further approved by the Ethics Committee of the Ludwig Maximilian University in Munich, Germany.

## Author contributions

Conception and experimental design: AHo, CG, and KH. Laboratory work and data generation: AH, NPi. Analysis and interpretation of the data: AHo, LR, OB, NPi, RW, CG and KH. Peptide Array design: GP and CG. Clinical trial conduct and management: SJ, MC, AHe, LM, FM, AJ, EV, L-AE, HK, SR-N, PP, SN, JD, NPr, SF, RS, MR, JW, SC, PM,EL, CN, AK, MH. Supervision: RW, CG and KH. Funding acquisition: MH and CG. Manuscript: AHo, CG and KH drafted the manuscript. All authors contributed to the article and approved the submitted version.
