# Supplementary material for: Systematic comparison of HIV-1 Envelope-specific IgG responses induced by different vaccination regimens: Can we steer IgG recognition towards regions of viral vulnerability?
Source: Front Immunol. 2023 Jan 9;13:1075606. doi: 10.3389/fimmu.2022.1075606 (PMC9891136; doi:10.3389/fimmu.2022.1075606)
Supplement: Supplementary file 1 [file DataSheet_1.docx]

Supplementary Material

**
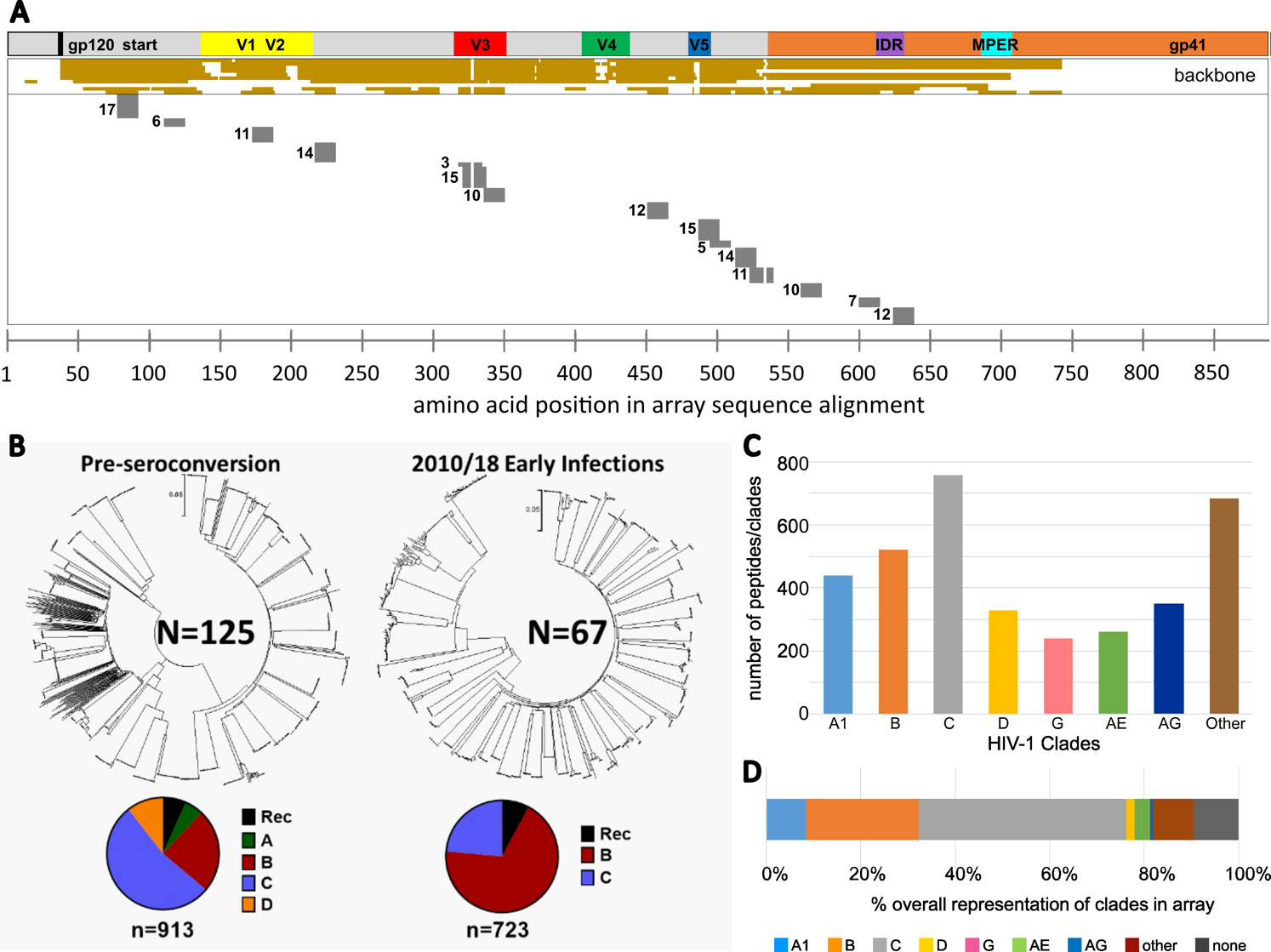
**

**Supp Fig 1: Peptide microarray design for the evaluation of HIV-1 Env-specific antibody responses.**

**A:** Representation of the alignment of all sequences included in the array. The backbone depicted in brown consist of 15mer peptides overlapping by 11 amino acids covering 10 full-length Env immunogen sequences included in the peptide array from the gp120start to the array amino acid position 743 (corresponding to HXB2 aa718) in gp41. Shown below in grey are the additional 162 15mer peptides covering frequently occurring additional antigenic variant sequences for 15 immuno-dominant Env regions. **B:** Selection of frequently occurring additional antigenic variant sequences for immuno-dominant Env regions. All existing pre-seroconversion (n=913) and recent (n=723) HIV infection sequences from 192 subjects were obtained from the HIV database (www.hiv.lanl.gov, accession in May 2018). The pie charts show the subtype distribution of these Env sequences. The phylogenetic trees visualise their relationship. These sequences were interrogated to identify the most frequently occurring molecular forms for the 15 regions. **C:** The 1034 peptides in the microarray stratified by clade. **D:** Percentage overall representation of HIV-1 clades by 15mer peptides in the microarray. Single peptides can be representative of more than one clade (C/D).

**Supplementary Figure 2: Above average V3 detection is not linked to below average V2 detection or below average overall Env reactivity in individual vaccinees.**

Relationship between V3 responses and (**A**) V2 responses (TMV02, TMV02+CN54gp140 and RV144) and (**B**) total sum of detected epitopes (including all analysed groups). Each line connects the values of an individual vaccinee. FI values of all vaccine trials were normalised by calculating the median responses for each epitope (max FI) and the deviation of each individual to the median. The x-axis (0 deviation) indicates the median. Colour coding in (**A**) indicates the different groups (blue = RV144, red = TMV02 and brown = TMV02 +CN54gp140).

**Supplementary Figure 3: Positive effect of DNA priming in RV172.**

Comparison of total IgG responses between the Ad5 only RV172 group (orange) and the DNA primed RV172+DNA group (yellow). **A**: The number of peptides detected per patient, indicating the breadth of linear IgG recognition. **B**: Sum of all max FI values per patient indicating the overall strength of HIV-1 Env detection. Statistical significance was evaluated by Mann-Whitney u-test.

**Supplementary Table1: Detailed Information on immunogens**

**Supplementary Table 2: Mean FI values and frequency of responders (FOR) of the IDRs**

**NOTE:** Boldface type indicated dominant recognition, defined by a detection FOR of at least 60% and mean FI values of the top 15%. Mean FI values were calculated if FOR>25%.

IDR, immunodominant region; FOR, frequency of Responders; Env, Envelope

**Supplementary Table 3: Immunogen sequences of the IDRs**


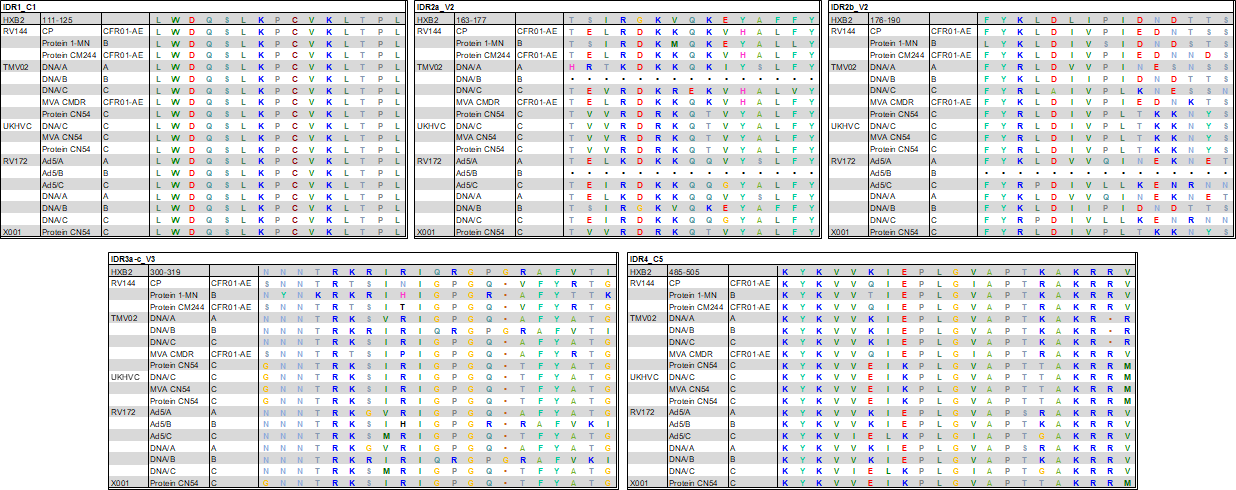


For each region, the HXB2 site with corresponding representative sequence is indicated, as well as the sequence variants of each Env immunogen included in the vaccination regimen of one of the 5 analysed trials.
